# An Extremely Sparse Tomography Reconstruction of a Multispectral Temperature Field without Any a Priori Knowledge

**DOI:** 10.3390/s24165264

**Published:** 2024-08-14

**Authors:** Xuan Zhang, Yan Han

**Affiliations:** 1 School of Information and Communication Engineering, North University of China, Taiyuan 030051, China; zhangxuanjohn@163.com; 2 Shanxi Key Laboratory of Signal Capturing & Processing, North University of China, Taiyuan 030051, China; 3 State Key Laboratory of Dynamic Testing Technology, North University of China, Taiyuan 030051, China

**Keywords:** image reconstruction, optical tomography, sparse projection, temperature inversion

## Abstract

When undertaking optical sparse projection reconstruction, the reconstruction of the tested field often requires the utilization of a priori knowledge to compensate for the lack of information due to the sparse projection angle. In order to reconstruct the radiation field of unknown materials or in situations where a priori knowledge cannot be obtained, this paper proposes an extremely sparse tomography multispectral temperature field reconstruction algorithm that analyzes the similarity (the similarity here compares and calculates the Euclidean distance of the spectral emissivity values at various wavelengths between different spectral curves) of radiation characteristics of materials under the same pressure and concentration but different temperature, describes the similarity between the radiation information of the tested field using the dynamic time warping (DTW) algorithm, and uses the similarity sum of the radiation information among the subregions of the temperature field as the optimization objective. This is combined with the equation-constrained optimization algorithm and multispectral thermometry to establish the statistical law between the missing information and finally realize the reconstruction of the temperature field. Simulation experiments show that, without any a priori knowledge, the method in this paper can realize reconstruction of the temperature field with an accuracy of 1.53–12.05% under two projection angles and has fewer projection angles and stronger robustness than other methods.

## 1. Introduction

Optical tomography is a diagnostic technology that does not interfere with the tested field, and it has shown great advantages in physical measurement, temperature field measurement, plasma diagnosis, and other aspects [1,2,3,4]. In order to overcome the assumption that the pressure is constant, uniform and known during temperature measurement, Weiwei Cai et al. proposed a new optical tomography technique. This technique realizes the synchronous distribution of temperature and concentration by optimizing the cost function between the reconstructed region and the actual region and obtains the accurate spectrum by introducing the regularization term, which is the mean absolute deviation between the parameters of a region and the adjacent region. Simulation experiments on representative flame phantoms have confirmed the validity and robustness of the method [5]. Min-Gyu Jeon et al. have recently proposed three new reconstruction algorithms, MART (multiplicative algebraic reconstruction technique), SART (simultaneous algebraic reconstruction technique), and SMART (simultaneous multiplicative algebraic reconstruction technique) for CT-TDLAS (computed tomography-tunable diode laser absorption spectroscopy). By improving the contribution of the relaxation factor in the ART (algebraic reconstruction technique) algorithm to iteration quantity, the accuracy of temperature field reconstruction results was improved. By comparing the reconstruction results with the previous algorithms, the performance of the new algorithm was verified. The experimental results show that SMART has the fastest reconstruction speed, converges after 50 iterations, and has the highest accuracy, up to 1.4% [6]. To provide high-speed imaging technology for fuel mixing, Paul Wright et al. developed a low-noise optoelectronic system coupled with an optical access layer (OPAL) to achieve tomography of 27 angles of highly dynamic chemical reaction processes through offline processing under the condition of uniform fuel distribution [7]. Chang Liu et al. used a fan-beam laser instead of a parallel beam laser to simplify the optical structure of TDLAS. Meanwhile, combined with the onion peel deconvolution algorithm, TDLAS can be easily applied in actual flame measurement, improving its accuracy and robustness. Numerical simulation and experiments under 40 projection angles demonstrate the effectiveness of the proposed method [8]. To solve the problem of the slow speed of algebraic reconstruction algorithms in clinical applications, Klaus Mueller et al. proposed a scheme that extends the precision of a given framebuffer by 4 bits, using the color channels. With this extension, a 12-bit framebuffer delivers useful reconstructions for 0.5% tissue contrast, while an 8-bit framebuffer requires 4%. Finally, 3D algebraic fast reconstruction was realized under 80 projection angles [9].

The above studies show the advantages of optical tomography in performance. In general, it is not possible to obtain test data from all angles. For most optical computed tomography (OpCT) methods, due to environmental limitations and the necessity for complex test equipment (such as beam deflection, interferometry, light emission computed tomography (LECT), and emission spectral tomography (EST)), it is not possible to obtain test data from all angles. The amount of data for each angle is limited (the data are incomplete) [10,11]. Therefore, a CT algorithm with limited data is needed in OpCT reconstruction. When the maximum entropy (ME) algorithm is used for tomography reconstruction in the case of limited projection views, it is difficult to accurately reconstruct test fields with a complex distribution. In order to solve this problem, Xiong Wan et al. proposed a fusion entropy algorithm, which combines the maximum entropy and cross-entropy (CE) and works with OpCT to achieve tomography reconstruction under six projection angles. Numerical simulation results showed that the proposed algorithm has high reconstruction accuracy in both symmetric and asymmetric fields [12]. Haimiao Zhang et al. proposed a new network for sparse angle CT image reconstruction, which only learns the experience part of the traditional network and keeps the rest intact. This method can greatly reduce the design of trainable parameters while maintaining the superior performance of traditional networks in CT image reconstruction. Simulation and experimental verification show that the network can achieve high-precision CT image reconstruction only under 15 projection angles [13]. Yiqing Gao et al. proposed a new chromatographic reconstruction method for the three-dimensional plasma temperature field. This method uses the camera to obtain the spectral information of the temperature field, adopts the improved CT reconstruction algorithm, and combines the maximum entropy and least square methods to process the spectral data, finally realizing the three-dimensional temperature field reconstruction by using prior knowledge. Experimental results show that this method has high accuracy and real-time performance under 2–4 projection angles [14].

In the process of optical tomography reconstruction, although the above research methods can realize the reconstruction of the field with a lower projection angle, the premise is that prior knowledge must be used to make up for the information loss caused by the sparse projection angle. In the combustion temperature measurement of complex structural materials, including special energy materials such as explosives, their composition materials are complex and generally consist of a variety of materials with different ratios; their radiation characteristics are also different and have different ratios, which makes them difficult to describe in the context of spectral characteristics of materials at a static state. Moreover, in the combustion process, the spectral characteristics of the material will change dynamically, and even if the spectral characteristics can be measured in static, they will also be very different from the actual combustion. Therefore, in the process of radiation field reconstruction of unknown materials or without prior knowledge, it is difficult to achieve optical tomography reconstruction by using the above method. To solve the above problems, this paper proposes an extremely sparse tomography multispectral temperature field reconstruction algorithm. When analyzing the similarity of radiation characteristics of materials at different temperatures, a dynamic time warping (DTW) algorithm is used to describe the similarity between radiation information of the tested field and the sum of the similarity of radiation information between sub-regions of the temperature field is taken as the optimization objective. The statistical rule of missing information is established by combining the equation constraint optimization algorithm and multispectral thermometry technology, which indirectly compensates for the lack of information caused by the sparse projection angle, and finally, the reconstruction of the temperature field can be realized.

## 2. Basic Principles

**Figure 1:**
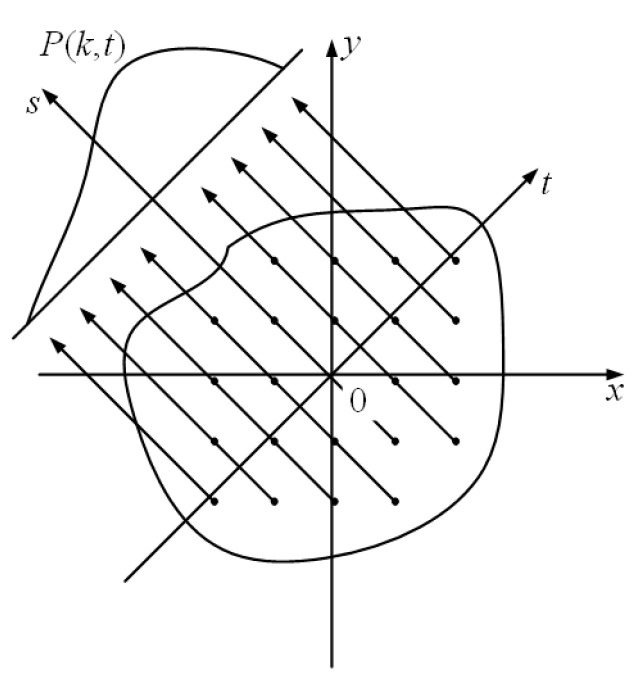
The projection process of the CT algorithm at a certain angle of the measured field.

The basic idea behind the optical tomography reconstruction algorithm is to use the detector to collect the radiation projection distribution of the tested field at an appropriate angle and finally reconstruct the field according to the projection distribution; the reconstruction of the field will also be the reconstruction of the original function from the line integral. As shown in Figure 1, $f\left(x,y\right)$ is the tested field, *x*-*y* is the right-angle coordinate system, *s*-*t* is the coordinate system after rotating the *x*-*y* coordinate axis along the counterclockwise direction by an angle of *θ_k_*, and the projection *P*(*k*,*t*) of *f*(*x*,*y*) along the *k*th angle is expressed by the following equation:

(1) $$P\left(k,t\right)=\int_{\left(t,\theta_{k}\right)line}f\left(x,y\right)ds=\int_{-\infty }^{\infty }f\left(t\cos\theta_{k}-s\sin\theta_{k},t\sin\theta_{k}+s\cos\theta_{k}\right)ds,$$

where $P\left(k,t\right)$ is the projection data, and the transformation relationships between the two coordinate systems are $x=t\cos\theta_{k}-s\sin\theta_{k}$ and $y=t\sin\theta_{k}+s\cos\theta_{k}$.

**Figure 2:**
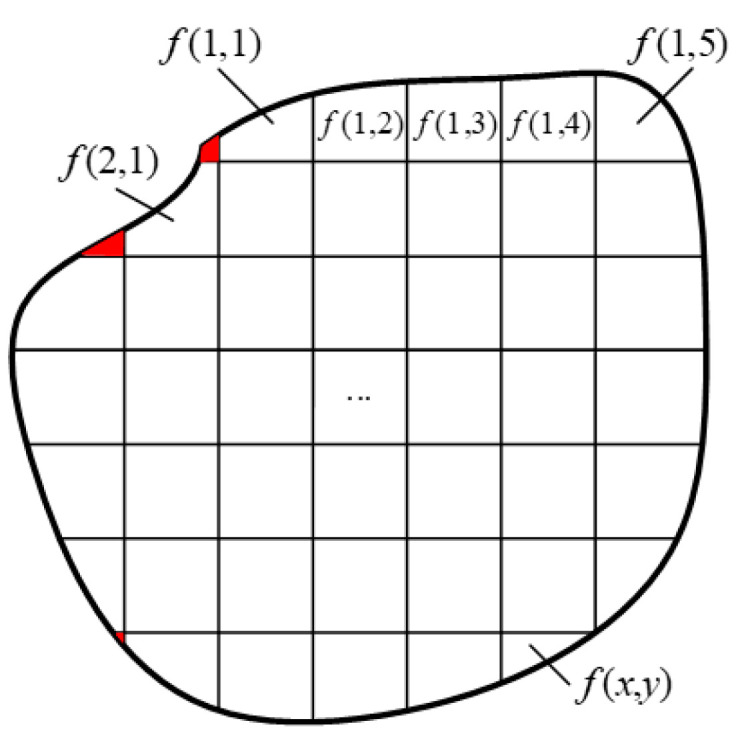
Schematic diagram of the tested field. The red area in the figure has little influence on the reconstruction and is neglected.

For the convenience of measurement and calculation, the tested field is divided into subregions and numbered, as shown in Figure 2, and the red area in the figure has little influence on the reconstruction and is neglected. The reconstruction of the to-be-measured field and the representation of the projection data are changed from line integration to the form of summation, and Equation (1) is transformed into the following equation:

(2) $$P\left(k,t\right)=\sum_{s=-\infty }^{\infty }f\left(t\cos\theta_{k}-s\sin\theta_{k},t\sin\theta_{k}+s\cos\theta_{k}\right),$$

In mathematics, it is known that in a case with few projection angles, the reconstruction of the tested field with the projection data is converted into a numerical optimization problem under equational constraints. The basic form of the equationally constrained problem is as follows:

(3) $$\left\{\begin{array}{c} minF\\ s.t.c_{i}=0\left(i=1,2,\ldots ,n\right) \end{array}\right.,$$

where *F* denotes the objective function to be optimized, and *c_i_* denotes the equation constraints.

### 2.1. Objective Function

**Figure 3:**
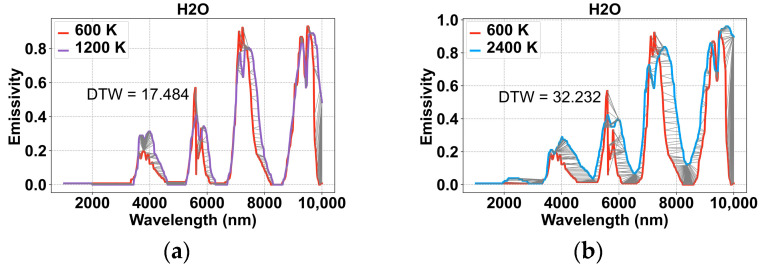

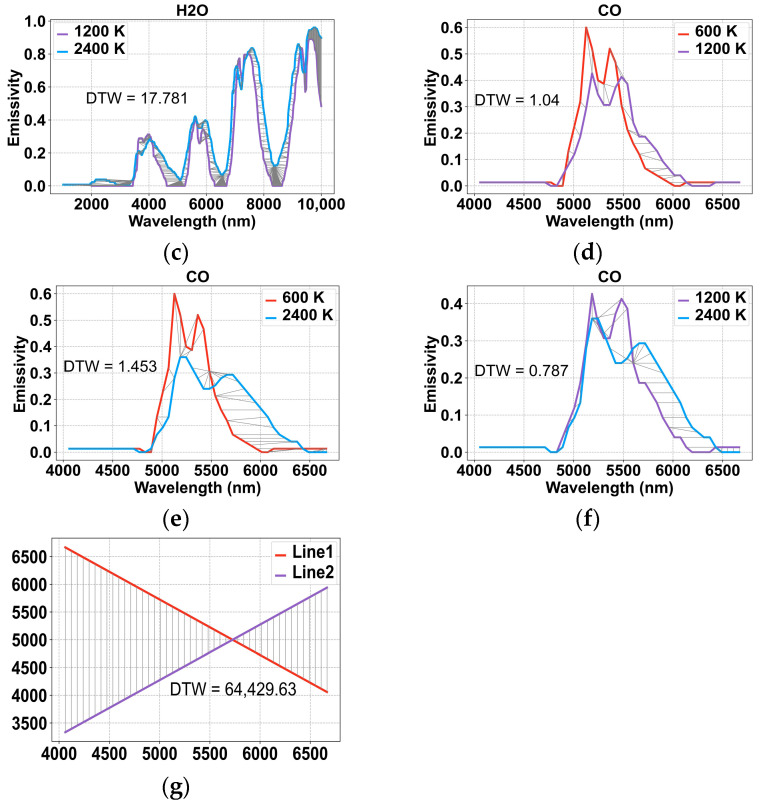
The similarity between different radiation characteristics: (**a**) The similarity of radiation characteristics of H_2_O at 600 K and 1200 K; (**b**) the similarity of radiation characteristics of H_2_O at 600 K and 2400 K; (**c**) the similarity of radiation characteristics of H_2_O at 1200 K and 2400 K; (**d**) the similarity of radiation characteristics of CO at 600 K and 1200 K; (**e**) the similarity of radiation characteristics of CO at 600 K and 2400 K; (**f**) the similarity of radiation characteristics of CO at 1200 K and 2400 K; (**g**) the similarity of radiation characteristics of uncorrelated curves.

In order to reconstruct the temperature field during fuel combustion, the radiative properties of different materials need to be analyzed. The radiative properties of CO and H_2_O published by NASA [15] are shown in Figure 3, in which the spectral emissivities of CO and H_2_O in the 2000–10,000 nm band show very strong nonlinear characteristics and a large amount of CO and H_2_O is accompanied by chemical fuel combustion, explosive detonation, rocket engine propellant combustion, and other processes. Therefore, CO and H_2_O were studied as examples.

From Figure 3, it can be seen that although the radiation characteristics of the same material in the temperature range of 600–2400 K are not exactly the same, and there are certain translations or distortions, their general trends are the same. The DTW algorithm is an accurate method to calculate the similarity between different nonlinear sequences; therefore, in order to explore the relationship, the DTW value is used to describe the similarity between different radiation characteristics of the same material, and the results are shown in Figure 3. The similarity here compares and calculates the Euclidean distance of the spectral emissivity values at various wavelengths between different spectral curves. It’s a one-dimensional number.

Among them, Figure 3a to 3c represent the DTW similarity between different radiative properties of H_2_O in the range of 600 K–2400 K, Figure 3d to 3f represent the DTW similarity between different radiative properties of CO, and Figure 3g represents the DTW values between the two irrelevant curves. As can be seen from Figure 3, the DTW values of the same material at different temperatures are very small, while the DTW values of the irrelevant curves are very large, and this phenomenon indirectly verifies the feasibility of adopting the DTW to describe the similarity of the material’s radiation properties.

**Figure 4:**
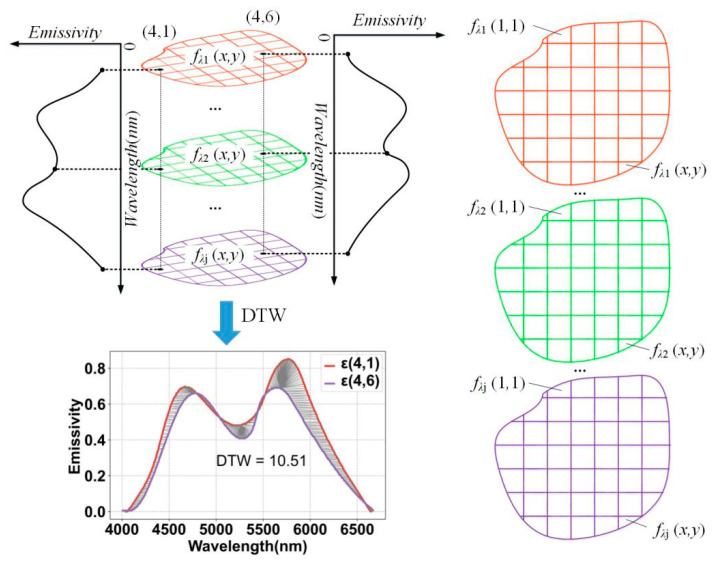
The field of multi-band radiation.

It can be seen from the above analysis that the same material exhibits similar radiation characteristics at different temperatures. Therefore, in order to utilize the spectral information in the temperature field to make up for the lack of information in the less-angled projection data, the temperature field in Figure 2 is divided into a multi-band radiation field, as shown in Figure 4, by adding in the spectral separation technique.

From Figure 4, it can be seen that the temperature field $f\left(x,y\right)$ is separated into $f_{\lambda_{1}}\left(x,y\right)$, $f_{\lambda_{2}}\left(x,y\right)$, ···, and $f_{\lambda_{j}}\left(x,y\right)$ at *λ*_1_, *λ*_2_, ···, and *λ_j_* wavelengths, and the radiative properties of the temperature field at (4,1) and (4,6) are similar to each other, with a DTW value of 10.51. Similarly, the radiative properties of the temperature field between other subregions are also similar to each other, i.e., in the case that the spectral emissivities are unknown in each part of the radiative field, the sum of the DTW values of the subregions exists as an extreme value, as shown in the following equation:

(4) $$\begin{array}{c} DTW\left(\varepsilon \left(1,1\right),\varepsilon \left(1,2\right)\right)+DTW\left(\varepsilon \left(1,1\right),\varepsilon \left(1,3\right)\right)+\ldots +DTW\left(\varepsilon \left(1,1\right),\varepsilon \left(x,y\right)\right)+\\ DTW\left(\varepsilon \left(1,2\right),\varepsilon \left(1,3\right)\right)+\ldots +DTW\left(\varepsilon \left(1,2\right),\varepsilon \left(x,y\right)\right)+\ldots +DTW(\varepsilon (x,y-\\ 1),\varepsilon \left(x,y\right))+value=0, \end{array}$$

where $DTW\left(\varepsilon \left(1,1\right),\varepsilon \left(1,2\right)\right)$ is the DTW value of the radiation characteristics at the subregions (1,1) and (1,2), $\varepsilon \left(x,y\right)$ is the radiation characteristics at the subregion (*x*,*y*), and is denoted by $\left(\varepsilon_{\lambda_{1}}\left(x,y\right),\varepsilon_{\lambda_{2}}\left(x,y\right){,\ldots ,\varepsilon }_{\lambda_{j}}\left(x,y\right)\right)$, and *value* is the extreme value of the DTW sum of the subregions. It can be seen from [16] that $\varepsilon \left(x,y\right)$ can be represented by the following formula:

(5) $$\varepsilon \left(x,y\right)=\left(\frac{f_{\lambda_{1}}\left(x,y\right){\cdot e}^{\frac{C_{2}}{\lambda_{1}T_{\lambda_{1}}\left(x,y\right)}}}{f_{b}\left(x,y\right){\cdot e}^{\frac{C_{2}}{\lambda_{1}T_{\lambda_{1}b}\left(x,y\right)}}},\frac{f_{\lambda_{2}}\left(x,y\right){\cdot e}^{\frac{C_{2}}{\lambda_{2}T_{\lambda_{2}}\left(x,y\right)}}}{f_{b}\left(x,y\right){\cdot e}^{\frac{C_{2}}{\lambda_{2}T_{\lambda_{2}b}\left(x,y\right)}}},\ldots ,\frac{f_{\lambda_{j}}\left(x,y\right){\cdot e}^{\frac{C_{2}}{\lambda_{j}T_{\lambda_{j}}\left(x,y\right)}}}{f_{b}{\left(x,y\right)\cdot e}^{\frac{C_{2}}{\lambda_{j}T_{\lambda_{j}b}\left(x,y\right)}}}\right),$$

where $f_{\lambda_{j}}\left(x,y\right)$ denotes the radiation of the position (*x*,*y*) at temperature $T_{\lambda_{j}}\left(x,y\right)$ and wavelength $\lambda_{j}$, $T_{\lambda_{j}}\left(x,y\right)$ indicates the temperature to be measured, $f_{b}\left(x,y\right)$ denotes the radiation of the blackbody furnace at temperature $T_{\lambda_{j}b}\left(x,y\right)$, and $T_{\lambda_{j}b}\left(x,y\right)$ represents the reference temperature in the mathematical model based on the reference temperature in the multispectral temperature measurement method. The wavelength is $\lambda_{j}$, and the second radiation constant, *C*_2_, is used in physics to describe the properties of blackbody radiation.

Substituting Equation (5) into $DTW\left(\varepsilon \left(1,1\right),\varepsilon \left(1,2\right)\right)$ gives

(6) $$DTW\left(\varepsilon \left(1,1\right),\varepsilon \left(1,2\right)\right)=\sqrt{\sum_{l=1}^{j}{\left(\frac{f_{\lambda_{l}}\left(1,1\right){\cdot e}^{\frac{C_{2}}{\lambda_{l}T_{\lambda_{l}}\left(1,1\right)}}}{f_{b}\left(1,1\right){\cdot e}^{\frac{C_{2}}{\lambda_{l}T_{\lambda_{l}b}\left(1,1\right)}}}-\frac{f_{\lambda_{l}}\left(1,2\right){\cdot e}^{\frac{C_{2}}{\lambda_{l}T_{\lambda_{l}}\left(1,2\right)}}}{f_{b}\left(1,2\right){\cdot e}^{\frac{C_{2}}{\lambda_{l}T_{\lambda_{l}b}\left(1,2\right)}}}\right)}^{2}},$$

From Equation (4), the objective function of the equationally constrained optimization problem is

(7) $$\begin{array}{c} F=DTW\left(\varepsilon \left(1,1\right),\varepsilon \left(1,2\right)\right)+DTW\left(\varepsilon \left(1,1\right),\varepsilon \left(1,3\right)\right)+\ldots +DTW\left(\varepsilon \left(1,1\right),\varepsilon \left(x,y\right)\right)+\\ DTW\left(\varepsilon \left(1,2\right),\varepsilon \left(1,3\right)\right)+\ldots +DTW\left(\varepsilon \left(1,2\right),\varepsilon \left(x,y\right)\right)+\ldots +DTW\left(\varepsilon \left(x,y-1\right),\varepsilon \left(x,y\right)\right), \end{array}$$

**Figure 5:**
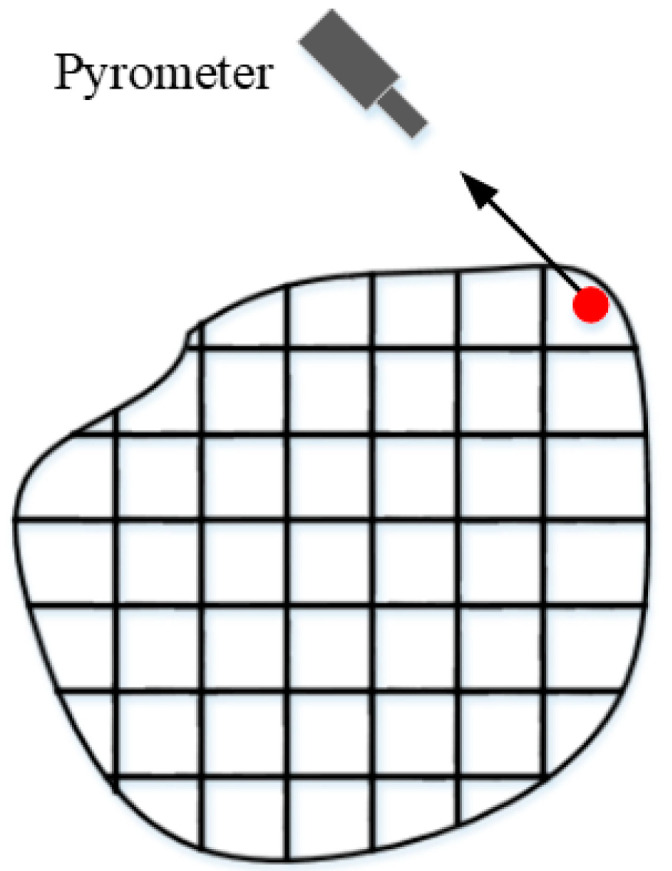
The acquisition method for reference radiation. The red dots indicate the reference radiation region. The black device indicates a pyrometer, which is composed of the telescope system, spectral dispersion system, and photoelectric detection system, and can produce the linear spectrum within the working spectrum range of the target and ensure the accurate acquisition of the spectral information of the temperature field.

Analyzing Equation (7), it can be seen that, assuming that the testing field $f\left(x,y\right)$ is divided into m subregions, the objective function *F* contains m! term DTW values, and when m reaches a certain value, the optimization of the objective function requires excellent arithmetic support. In order to improve the efficiency of the algorithm and to prevent the dependence of the algorithm on the computer’s arithmetic power, the objective function is simplified: the suitable subregion in the radiation field is selected as the reference radiation area so that the other subregions are, respectively, calculated with the reference radiation area for the DTW and summed up. After simplification, the objective function becomes

(8) $$F=DTW\left(\varepsilon \left(1,1\right),\varepsilon_{re}\right)+DTW\left(\varepsilon \left(1,2\right),\varepsilon_{re}\right)+\ldots +DTW\left(\varepsilon \left(x,y\right),\varepsilon_{re}\right),$$

where $\varepsilon_{re}$ denotes the reference radiation. The acquisition of the reference radiation in Equation (8) and the selection of the reference radiation area are shown in Figure 5.

As can be seen from Figure 5, using the pyrometer in [17], any subregion at the edge of the tested field at a suitable angle can be taken as the radiation reference area. The method of obtaining reference radiation is relatively flexible and not fixed. The principle is that after dividing the subregion, the spectrum of the target band of a certain subregion can be detected by using the appropriate angle, and the reference radiation can be solved by using the obtained spectrum. No matter what the geometry of the temperature field, this principle is relatively easy to achieve. Combining with the thermometry method in [16], the reference radiation is given by the following equation:

(9) $$\varepsilon_{re}=\left(\frac{f_{\lambda_{1}}{\cdot e}^{\frac{C_{2}}{\lambda_{1}T_{\lambda_{1}}}}}{f_{b}{\cdot e}^{\frac{C_{2}}{\lambda_{1}T_{\lambda_{1}b}}}},\frac{f_{\lambda_{2}}{\cdot e}^{\frac{C_{2}}{\lambda_{2}T_{\lambda_{2}}}}}{f_{b}{\cdot e}^{\frac{C_{2}}{\lambda_{2}T_{\lambda_{2}b}}}},\ldots ,\frac{f_{\lambda_{j}}{\cdot e}^{\frac{C_{2}}{\lambda_{j}T_{\lambda_{j}}}}}{f_{b}{\cdot e}^{\frac{C_{2}}{\lambda_{j}T_{\lambda_{j}b}}}}\right),$$

The objective function of the equationally constrained optimization problem can be obtained by substituting Equation (9) into Equation (8).

### 2.2. Constraints

**Figure 6:**
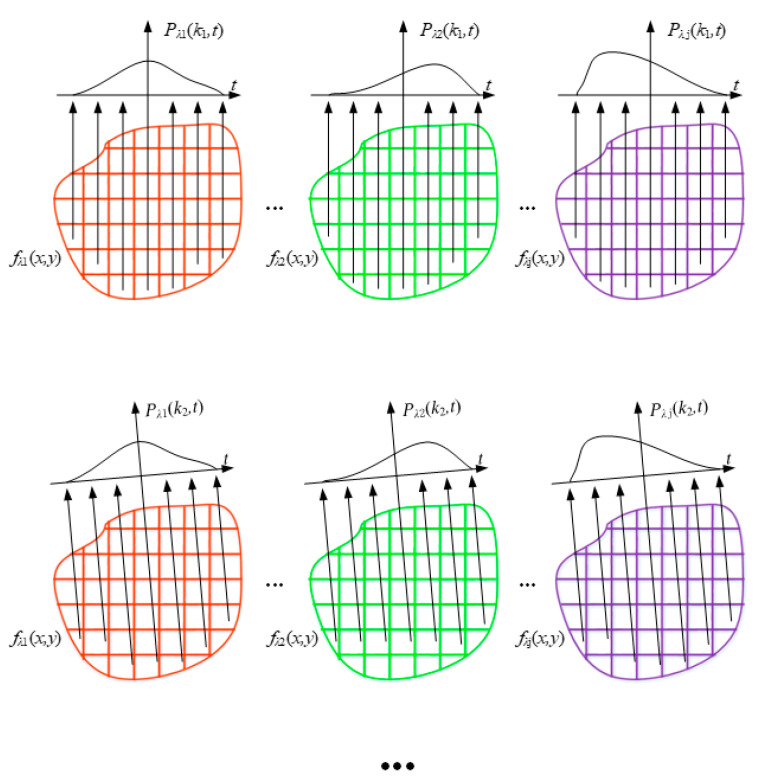
The projection data of radiation fields $f_{\lambda_{1}}\left(x,y\right)$, $f_{\lambda_{2}}\left(x,y\right)$, ···, and $f_{\lambda_{j}}\left(x,y\right)$ at different angles. The red, green, and purple regions represent the temperature fields $f_{\lambda_{1}}\left(x,y\right)$, $f_{\lambda_{2}}\left(x,y\right)$, and $f_{\lambda_{j}}\left(x,y\right)$. The arrow indicates the direction of the projection. The curve on the axis represents the spectral curve detected at a projection angle.

In order to limit the optimization range of the objective function, it is necessary to constrain the range of values of the radiation (i.e., unknowns) of the field; the projection data of the multi-band radiation field are shown in the following Figure 6.

From Figure 6, when the projection angle *θ_k_* takes the value of *k*_1_, the projection data of the field at the wavelengths of *λ*_1_, *λ*_2_, …, and *λ_j_* are

(10) $$\left\{\begin{array}{c} P_{\lambda_{1}}\left(k_{1},t\right)=\sum_{s=-\infty }^{\infty }f_{\lambda_{1}}\left(t\cos k_{1}-s\sin k_{1},t\sin k_{1}+s\cos k_{1}\right)\\ P_{\lambda_{2}}\left(k_{1},t\right)=\sum_{s=-\infty }^{\infty }f_{\lambda_{2}}\left(t\cos k_{1}-s\sin k_{1},t\sin k_{1}+s\cos k_{1}\right)\\ \begin{array}{c} \ldots \\ P_{\lambda_{j}}\left(k_{1},t\right)=\sum_{s=-\infty }^{\infty }f_{\lambda_{j}}\left(t\cos k_{1}-s\sin k_{1},t\sin k_{1}+s\cos k_{1}\right) \end{array} \end{array}\right.,$$

Similarly, the multi-band radiation projection data *P*(*k*_1_,*t*), *P*(*k*_2_,*t*), … can be obtained by changing *θ_k_* at different angles. The projection data are the key information for the reconstruction of the field, which constrains the relationship between the spatial information in the field and the radiative information, constituting the constraints shown in Equation (11).

(11) $$\left\{\begin{array}{c} \sum_{s=-\infty }^{\infty }f_{\lambda_{1}}\left(t\cos k_{1}-s\sin k_{1},t\sin k_{1}+s\cos k_{1}\right)-P_{\lambda_{1}}\left(k_{1},t\right)=0\\ \sum_{s=-\infty }^{\infty }f_{\lambda_{2}}\left(t\cos k_{1}-s\sin k_{1},t\sin k_{1}+s\cos k_{1}\right){-P}_{\lambda_{2}}\left(k_{1},t\right)=0\\ \begin{array}{c} \ldots \\ \begin{array}{c} \sum_{s=-\infty }^{\infty }f_{\lambda_{j}}\left(t\cos k_{1}-s\sin k_{1},t\sin k_{1}+s\cos k_{1}\right){-P}_{\lambda_{j}}\left(k_{1},t\right)=0\\ \sum_{s=-\infty }^{\infty }f_{\lambda_{1}}\left(t\cos k_{2}-s\sin k_{2},t\sin k_{2}+s\cos k_{2}\right){-P}_{\lambda_{1}}\left(k_{2},t\right)=0\\ \begin{array}{c} \sum_{s=-\infty }^{\infty }f_{\lambda_{2}}\left(t\cos k_{2}-s\sin k_{2},t\sin k_{2}+s\cos k_{2}\right)-P_{\lambda_{2}}\left(k_{2},t\right)=0\\ \ldots \\ \begin{array}{c} \sum_{s=-\infty }^{\infty }f_{\lambda_{j}}\left(t\cos k_{2}-s\sin k_{2},t\sin k_{2}+s\cos k_{2}\right)-P_{\lambda_{j}}\left(k_{2},t\right)=0\\ \ldots \end{array} \end{array} \end{array} \end{array} \end{array}\right.,$$

After the above analysis, it can be seen that Equation (3) is transformed into the following formula:

(12) $$\left\{\begin{array}{c} minF=min(DTW\left(\varepsilon \left(1,1\right),\varepsilon_{re}\right)+DTW\left(\varepsilon \left(1,2\right),\varepsilon_{re}\right)+\ldots +DTW\left(\varepsilon \left(x,y\right),\varepsilon_{re}\right))\\ s.t.c_{i}=\begin{array}{c} \sum_{s=-\infty }^{\infty }f_{\lambda_{1}}\left(t\cos k_{1}-s\sin k_{1},t\sin k_{1}+s\cos k_{1}\right)-P_{\lambda_{1}}\left(k_{1},t\right)=0\\ \sum_{s=-\infty }^{\infty }f_{\lambda_{2}}\left(t\cos k_{1}-s\sin k_{1},t\sin k_{1}+s\cos k_{1}\right){-P}_{\lambda_{2}}\left(k_{1},t\right)=0\\ \begin{array}{c} \ldots \\ \begin{array}{c} \sum_{s=-\infty }^{\infty }f_{\lambda_{j}}\left(t\cos k_{1}-s\sin k_{1},t\sin k_{1}+s\cos k_{1}\right){-P}_{\lambda_{j}}\left(k_{1},t\right)=0\\ \sum_{s=-\infty }^{\infty }f_{\lambda_{1}}\left(t\cos k_{2}-s\sin k_{2},t\sin k_{2}+s\cos k_{2}\right){-P}_{\lambda_{1}}\left(k_{2},t\right)=0\\ \begin{array}{c} \sum_{s=-\infty }^{\infty }f_{\lambda_{2}}\left(t\cos k_{2}-s\sin k_{2},t\sin k_{2}+s\cos k_{2}\right)-P_{\lambda_{2}}\left(k_{2},t\right)=0\\ \ldots \\ \begin{array}{c} \sum_{s=-\infty }^{\infty }f_{\lambda_{j}}\left(t\cos k_{2}-s\sin k_{2},t\sin k_{2}+s\cos k_{2}\right)-P_{\lambda_{j}}\left(k_{2},t\right)=0\\ \ldots \end{array} \end{array} \end{array} \end{array} \end{array} \end{array}\right.,$$

### 2.3. Reconstruction of the Field

**Figure 7:**
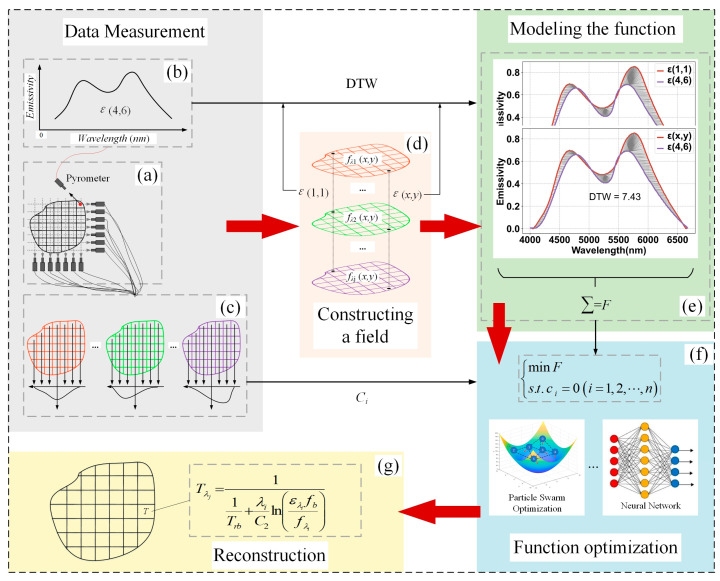
The process of temperature field reconstruction: (**a**) Data acquisition; (**b**) reference radiation acquisition; (**c**) projection data processing; (**d**) constructing a multi-band radiation field; (**e**) modeling the function; (**f**) function optimization; (**g**) reconstruction.

In order to achieve the sparse tomography reconstruction of multispectral temperature fields, the objective function of Equation (8) is optimized using the constraints of Equation (11), and the process is shown in Figure 7. The specific steps are as follows.

1 Data measurement: As shown in Figure 7a, a pyrometer is used to take any subregion at the edge of the field as the radiation reference area, and the reference radiation of the subregion is measured as shown in Figure 7b by combining the thermometry method in [16]. At the same time, multiple thermometry units are used to form a distributed network to collect the projection data from multiple angles of the field, which are then used as the constraints for the reconstruction of the field; the projection data are shown in Figure 7c.
2 Constructing a multi-band radiation field: construct the radiation fields $f_{\lambda_{1}}\left(x,y\right)$, $f_{\lambda_{2}}\left(x,y\right)$, ···, and $f_{\lambda_{j}}\left(x,y\right)$ shown in Figure 7d at *λ*_1_, *λ*_2_, ···, and *λ*_j_ wavelengths, respectively, divide them into the subregions shown in Figure 7d and number them.
3 Modeling the function: According to the thermometry method in [16], the radiation information in $f_{\lambda_{1}}\left(x,y\right)$, $f_{\lambda_{2}}\left(x,y\right)$, ···, and $f_{\lambda_{j}}\left(x,y\right)$ is used to establish the radiation characteristics of the subregion, and the results are shown in Figure 7e. The DTW values between the subregions and the reference radiation are calculated and summed to establish the function model *F* for the reconstruction of the field.
4 Function optimization: As shown in Figure 7f, the field reconstruction objective function is optimized by using the constraints of the equation obtained in step (1) above, and the function solution is judged to reach the optimal value according to the convergence condition of the algorithm; otherwise, it is returned to step (2) to optimize the radiation value of each band of the temperature field until the optimal solution is obtained. The optimization algorithm can be realized using gradient descent, particle swarm and neural network.
5 Reconstruction of the temperature field: The reconstruction of the temperature field is achieved by substituting the solution obtained from the function optimization and calculating the subregion temperature values using the thermometry method in [16]. This process is shown in Figure 7g.

After the above principle analysis, the theoretical abstraction of temperature field reconstruction can be completed, as well as the conversion to the equational constraint problem, and the reconstruction of the sparse tomography multispectral temperature field is realized.

## 3. Simulation Verification

### 3.1. Simulation Setup

**Figure 8:**
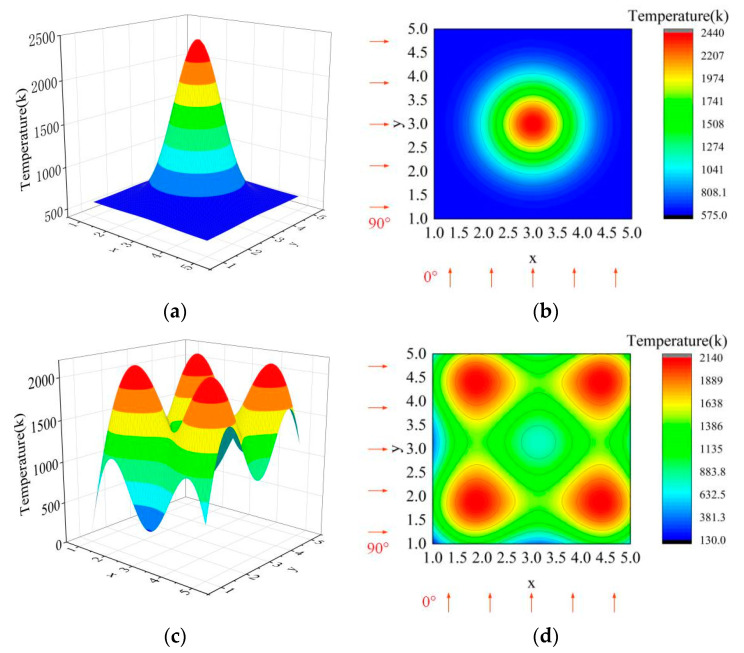
Tested temperature field: (**a**) The spatial distribution of the single-peak temperature field; (**b**) the temperature distribution of the single-peak temperature field; (**c**) the spatial distribution of the multi-peak temperature field; (**d**) the temperature distribution of the multi-peak temperature field. The arrows on the edge of the drawing indicate the direction and number of projections.

**Figure 9:**
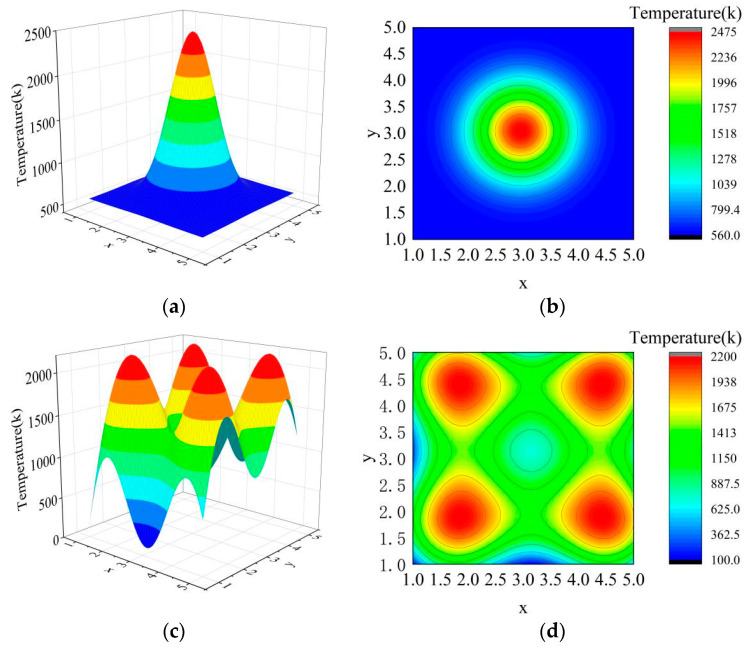
The results of temperature field reconstruction: (**a**) The result of the spatial distribution of the single-peak temperature field; (**b**) the result of the temperature distribution of the single-peak temperature field; (**c**) the result of the spatial distribution of the multi-peak temperature field; (**d**) the result of the temperature distribution of the multi-peak temperature field.

In order to verify the above theory of sparse tomography multispectral temperature field reconstruction, the temperature field in the temperature range of 600–2400 K in Figure 8 was taken as an example, and the above reconstruction method of the temperature field was utilized to complete the simulation verification. In the simulation, the square measured area is set up and divided into 5 × 5 sub-areas, and two sets of pyrometers are used to detect the projected data in the orthogonal direction, with five pyrometers in each set. The simulation is set up with single-peak as well as multi-peak temperature fields, as shown in Figure 8, where Figure 8a and Figure 9c show the spatial distribution of the temperature field, and Figure 8b and Figure 9d show the temperature distribution of the temperature field as well as the projection angle. The figures shown in the simulation process are the result of surface fitting. Data are processed in Python on a PC with an AMD Ryzen 5 5600G processor with Radeon Graphics 3.90 GHz and 16.00 GB of installed memory.

The spectral data used in the simulation were obtained from the spectral emissivity of CO in the 5066–5659 nm band published by NASA [15], and the specific values are shown in Table 1.

The radiation data of subregions of the temperature field in the simulation were calculated based on the temperature distribution in Figure 8 as well as the spectral data in Table 1, which are shown in the following equation.

(13) $$f_{\lambda_{i}}\left(x,y\right)=C_{1}\varepsilon_{\lambda_{i}}{\left(\pi \lambda_{i}^{5}e^{C_{2}/\lambda_{i}T\left(x,y\right)}\right)}^{-1},$$

where *C*_1_ is the first radiation constant. It describes the relationship between the energy density of blackbody radiation and the temperature. Combined with the projection direction in Figure 8, Equation (13) is substituted into Equation (2) to obtain the projection data required by the simulation. The specific values are shown in Table 2.

### 3.2. Simulation Results

In the simulation, the radiation characteristics at position (5,5) are selected as the reference radiation, and the projection data are substituted into the function model to realize the reconstruction of the temperature field; the reconstruction results are shown in Figure 9.

It can be seen from Figure 9 that the temperature field can be reconstructed under two projection angles by using the tomography reconstruction method in this paper, and the temperature distribution is basically consistent with the tested field. In order to evaluate the comparison results between Figure 8 and Figure 9 more effectively, the error distribution of the simulation results is calculated using the following formula.

**Figure 10:**
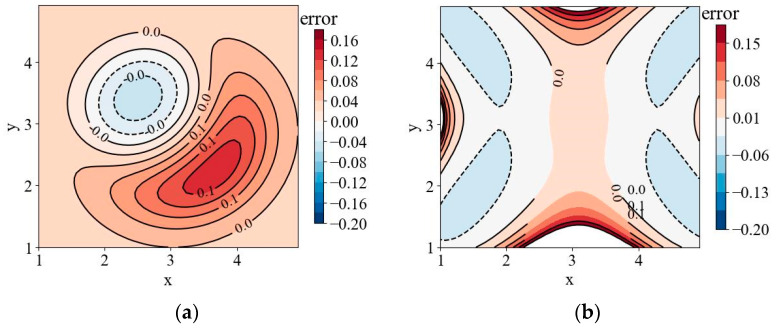
The error distribution of the simulation results: (**a**) The error distribution of the single-peak temperature field; (**b**) the error distribution of the multi-peak temperature field.

(14) errorx,y=Tx,y−T^x,yTx,y,

where $error\left(x,y\right)$ represents the error distribution of the simulation results, $T\left(x,y\right)$ represents the temperature distribution in Figure 8, and T^(x,y) represents the temperature distribution of the simulation results. The error distribution calculated by Equation (14) is shown in Figure 10.

Through an analysis of Figure 10a, it can be seen that there are some errors in the single-peak temperature field at the upper left and lower right. Through a careful comparison of Figure 8 and Figure 9, it can be seen that this phenomenon is caused by the shift of the peak center of the reconstructed temperature field, and its maximum error is 10.28%. According to the analysis of Figure 9 and Figure 10b, it can be seen that the reconstruction errors of the multi-peak temperature field are mainly concentrated at 0~500 °C and above 2000 °C. The errors of 0~500 °C are essentially distributed at the edge of the field, which is caused by inaccurate surface fitting and can be reduced by modifying the parameters of the surface fitting function. The error above 2000 °C is caused by the peak center deviation, and it needs to be eliminated by improving the spatial position reconstruction accuracy.

Through the above analysis, it can be seen that there are some errors in the reconstruction of the temperature field by the method used in this paper, but this does not affect its superiority in performance. In order to evaluate the accuracy of the method more accurately, the accurate error calculation formula shown in Equation (15) is used.

(15) Aver=∑x,yTx,y−T^x,yTmax×M×NMax=Tx,y−T^x,ymaxTmaxx,yRmse=∑x,yTx,y−T^x,y2∑x,yTx,y212,

In the formula, *Aver* represents the average error of the temperature field reconstruction, *M* and *N* represent the number of rows and columns of the field, *Max* represents the maximum error, and *Rmse* represents the root mean square error. The evaluation results of the reconstruction of the temperature field in Figure 9 obtained using the error calculation method of Equation (15) are shown in Table 3.

In the table, single-peak means only one temperature peak in the temperature field, while multi-peak means four temperature peaks in the temperature field. For details, please refer to Figure 9. As can be seen from Table 3, the error distribution of both single-peak and multi-peak temperature fields is Max>Rmse>Aver. This indicates that the temperature field reconstruction method used in this paper has the same reconstruction quality for temperature fields with different distributions; that is, the robustness of the method in this paper is strong. Overall, the proposed method can reconstruct the temperature field with a precision of 1.53–12.05%.

In order to evaluate the computational efficiency of the proposed method, 10 simulations were carried out on the temperature field reconstruction process above, and the function modeling time and temperature field reconstruction time of the proposed method were recorded; the respective values were averaged to obtain the function modeling time, 0.04 s, and the temperature field reconstruction time, 0.31 s.

### 3.3. Advantages of This Method

In order to explore the advantages of the proposed method in temperature field reconstruction, the performance of the proposed method was compared with that of [12,13,14], and the comparison results are shown in Table 4. ‘None’ indicates that no relevant data are published in [13].

As can be seen from Table 4, compared with other methods in [12,13,14], the method in this paper does not require any prior knowledge and can achieve temperature field reconstruction from just two angles. In addition, although the minimum error of the method in [14] is smaller than that of the proposed method, the maximum error is much higher than that of the proposed method, which indicates that the proposed method is more robust than the method in [14]. According to the known data, the proposed method in this paper has a lower time cost than the method in [13].

Through the above simulation, it can be seen that without any prior knowledge, the proposed method realizes the temperature field reconstruction with an accuracy of 1.53%–12.05% through the projection data from two angles, which has been verified as feasible and has certain advantages compared with other methods.

## 4. Discussion

### 4.1. The Influence of the Initial Value on the Reconstruction Results

**Figure 11:**
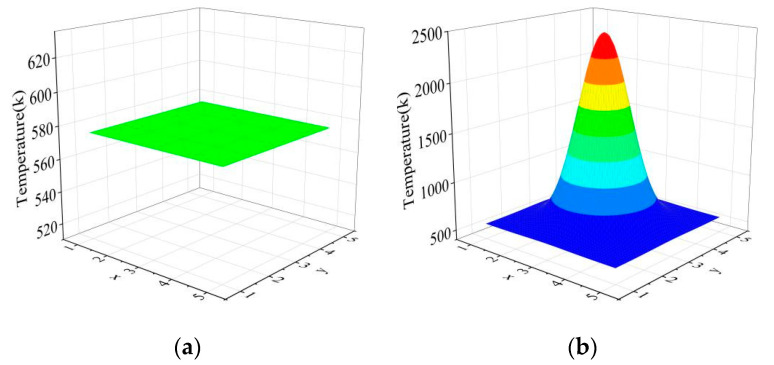
The reconstruction results of initial values of different iterations: (**a**) The reconstruction quality of the proposed method when the initial value of the subregion of the single-peak temperature field is the reference radiation; (**b**) the reconstruction quality of the proposed method when the initial value of the subregion of the single-peak temperature field is the mean value.

In order to further improve the method in future work, the factors that may affect the reconstruction quality are analyzed. Figure 11 shows the reconstruction quality of the proposed method when the initial values of the subregion of the single-peak temperature field are the reference radiation and the mean value of the projected data on the projection path.

As can be seen from the figure, the reconstruction quality of the temperature field varies greatly when the initial value of the temperature field is different. Figure 11a converges locally at a certain value; that is, the selection of the initial value of the field has a great factor on the reconstruction quality. To explore the maximum deviation between the initial value and the optimal result for the algorithm to be effective, we inversely extrapolated the optimal result to the initial input value and input 20%, 50%, 90%, 110%, 150%, 180%, and 200% of the initial input value into the function model. The maximum error of the optimization result is shown in the following Table 5.

As can be seen from the above table, the algorithm is effective when the initial input value is within the range of 40–160% of the best value. This phenomenon shows that although the method in this paper can reconstruct the temperature field without any prior knowledge, the question of how to select the initial value of the optimization function iteration will still be the focus of temperature field reconstruction in the future.

### 4.2. The Influence of the Angle between Two Projections on the Reconstruction Results

**Figure 12:**
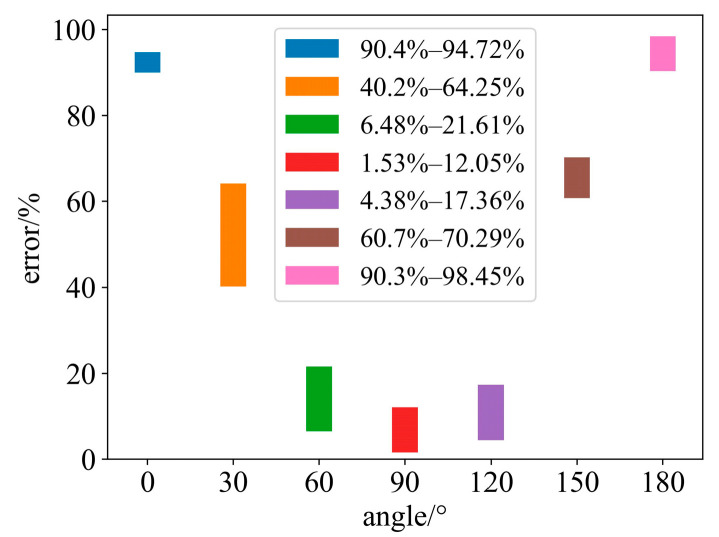
Reconstruction error of different projection angles. The error of angle 0° is 90.4–94.72%, the error of angle 30° is 40.2–64.25%, the error of angle 60° is 6.48–21.61%, the error of angle 90° is 1.53–12.05%, the error of angle 120° is 4.38–17.36%, the error of angle 150° is 60.7–70.29%, and the error of angle 180° is 90.3–98.45%.

In order to explore the influence of different angles between two projections on the reconstruction results, this paper set the angles as 0°, 30°, 60°, 90°, 120°, 150°, and 180° to acquire projection data for the measured field, and used the temperature field reconstruction method in this paper to process the acquired results. The reconstruction results are shown in the Figure 12 below.

As can be seen from the figure, when the angle between two projections is between 60° and 120°, the reconstruction error distribution is in a small range. When the angle exceeds this range, the reconstruction error will rapidly increase, and the error distribution is relatively concentrated, indicating that when the angle exceeds a certain value, the two projection data will become very redundant, and the algorithm in this paper will quickly converge in the place where the error is large. This phenomenon shows that the method should obtain as much information as possible from two projection data when selecting the projection angle so that the redundancy between data becomes smaller.

In addition, the symmetry of the temperature field is related to the reconstruction effect. When the field is symmetric, the 0° projection data are the same as the 180° projection data, and the 90° projection data are the same as the 270° projection data. The symmetry of the field helps to reduce the number of projections. However, this does not affect the superiority of this method without prior knowledge, and even if the number of projections is increased, the projections are still sparse. From the analysis in Figure 12, we can see that in the case of a large object shadowing a small object, we can still reconstruct the temperature field under the projection angle of 60°–120°, and 90° is not strict.

### 4.3. The Influence of the Surface Fitting Function on the Reconstruction Results

In order to explore the influence of different surface fitting functions on the reconstruction effect, this paper sets the surface fitting functions as 1, 2, and 3, carries out surface fitting for the reconstruction results, and defines the reduced temperature error and increased temperature error after replacing the fitting function as benefit/costs ratio (BCR), whose formula is shown below. The fitting results are shown in Table 6.

(16) $$BCR=\frac{\sum_{i=1}^{25}\left(T_{i}-T_{i}^{*}correct\right)}{\sum_{i=1}^{25}\left(T_{i}-T_{i}^{*}incorrect\right)},$$

where, $T_{i}$ represents the reconstruction temperature of each subregion without replacing the fitting function, $T_{i}^{*}correct$ represents the reconstruction temperature at which the error of each subregion becomes smaller after replacing the fitting function, and $T_{i}^{*}incorrect$ represents the reconstruction temperature at which the error of each sub-region becomes larger after replacing the fitting function. When the BCR is greater than 1, it is considered reasonable to replace the fitting function. Otherwise, it is unreasonable.

According to the analysis of Table 6, when the function is replaced from Poly2D to Cosine or Fourier2D, the error becomes larger and the BCR decreases, while when the function is replaced by Gaussian2D, the error becomes smaller and the BCR increases. Therefore, the error caused by surface fitting in the reconstruction process can be reduced by adjusting the function parameters.

## 5. Conclusions

An extremely sparse tomography multispectral temperature field reconstruction method is proposed in this paper. Without any a priori knowledge, the method can realize the reconstruction of the temperature field with an accuracy of 1.53–12.05% under two projection angles, and has fewer projection angles and stronger robustness than other methods. The function modeling time is 0.04 s, and the temperature field reconstruction time is 0.31 s. Moreover, this method can provide technical support for temperature field reconstruction when unknown materials or prior knowledge is not available.

## Figures and Tables

**Table 1:** The spectral emissivity of CO.

Temperature (K)	Wavelength (nm)										
	5066	5126	5185	5244	5304	5363	5422	5481	5541	5600	5659
600	0.32	0.6	0.52	0.4	0.39	0.52	0.47	0.31	0.21	0.16	0.12
1200	0.19	0.29	0.43	0.35	0.31	0.31	0.37	0.41	0.39	0.24	0.19
2400	0.13	0.28	0.36	0.36	0.32	0.28	0.24	0.24	0.25	0.28	0.29

**Table 2:** The projection data.

Projection (10^−11^ W·m^−2^·μm^−1^·sr^−1^)		Wavelength (nm)										
		5066	5126	5185	5244	5304	5363	5422	5481	5541	5600	5659
Single-peak	P(0°,1)	5.68	10.00	8.22	5.98	5.46	6.95	5.90	3.67	2.42	1.72	1.23
	P(0°,2)	4.26	6.97	7.35	5.51	4.79	5.24	5.20	4.45	3.61	2.24	1.64
	P(0°,3)	4.08	6.93	7.14	5.55	4.83	5.17	4.86	4.03	3.31	2.33	1.86
	P(0°,4)	4.26	6.97	7.35	5.51	4.79	5.24	5.20	4.45	3.61	2.24	1.64
	P(0°,5)	5.68	10.00	8.22	5.98	5.46	6.95	5.90	3.67	2.42	1.72	1.23
	P(90°,1)	5.68	10.00	8.22	5.98	5.46	6.95	5.90	3.67	2.42	1.72	1.23
	P(90°,2)	4.26	6.97	7.35	5.51	4.79	5.24	5.20	4.45	3.61	2.24	1.64
	P(90°,3)	4.08	6.93	7.14	5.55	4.83	5.17	4.86	4.03	3.31	2.33	1.86
	P(90°,4)	4.26	6.97	7.35	5.51	4.79	5.24	5.20	4.45	3.61	2.24	1.64
	P(90°,5)	5.68	10.00	8.22	5.98	5.46	6.95	5.90	3.67	2.42	1.72	1.23
Multi-peak	P(0°,1)	5.69	10.06	8.23	5.98	5.47	6.95	5.91	3.68	2.42	1.72	1.23
	P(0°,2)	5.70	10.08	8.25	5.99	5.48	6.97	5.92	3.68	2.43	1.73	1.23
	P(0°,3)	5.69	10.06	8.23	5.98	5.47	6.95	5.91	3.68	2.42	1.72	1.23
	P(0°,4)	5.70	10.08	8.25	5.99	5.48	6.97	5.92	3.68	2.43	1.73	1.23
	P(0°,5)	5.69	10.06	8.23	5.98	5.47	6.95	5.91	3.68	2.42	1.72	1.23
	P(90°,1)	5.69	10.06	8.23	5.98	5.47	6.95	5.91	3.68	2.42	1.72	1.23
	P(90°,2)	5.70	10.08	8.25	5.99	5.48	6.97	5.92	3.68	2.43	1.73	1.23
	P(90°,3)	5.69	10.06	8.23	5.98	5.47	6.95	5.91	3.68	2.42	1.72	1.23
	P(90°,4)	5.70	10.08	8.25	5.99	5.48	6.97	5.92	3.68	2.43	1.73	1.23
	P(90°,5)	5.69	10.06	8.23	5.98	5.47	6.95	5.91	3.68	2.42	1.72	1.23

**Table 3:** Error of temperature field reconstruction.

Temperature Field	*Aver* (%)	*Max* (%)	*Rmse* (%)
Single-peak	1.53	6.26	5.08
Multi-peak	2.1	12.05	3.99

**Table 4:** Advantages of this method.

Method	Prior knowledge	Error Distribution (%)	Projection Numbers	Modeling Time (s)	Reconstruction Time (s)
Textual method	×	1.53–12.05	2	0.04	0.31
Method in [12]	√	0.5–11.87	6	None	None
Method in [13]	√	None	15–180	None	0.4647–0.8479
Method in [14]	√	0.21–21.57	2–6	None	None

‘None’ indicates that there are no relevant data in the references.

**Table 5:** Results of different initial values.

The Ratio to the Best Initial Value	20%	40%	90%	110%	160%	180%	200%
Max error (%)	32.17	9.58	4.36	3.29	11.68	19.67	62.43

**Table 6:** Results of different surface fitting functions.

Function Name	Poly2D (Textual Function)	Cosine	Fourier2D	Gaussian2D
Error (%)	1.53–12.05	1.67–13.62	7.49–18.53	0.95–11.94
BCR	-	0.94	0.59	1.05

## Data Availability

The data presented in this study are available upon request from the corresponding author.
